# Clinical evaluation of autologous *gamma delta* T cell-based immunotherapy for metastatic solid tumours

**DOI:** 10.1038/bjc.2011.293

**Published:** 2011-08-16

**Authors:** A J Nicol, H Tokuyama, S R Mattarollo, T Hagi, K Suzuki, K Yokokawa, M Nieda

**Affiliations:** 1 Department of Medicine, Centre for Immune and Targeted Therapy, University of Queensland, Brisbane, 4120 Australia; 2 Fourth Department of Internal Medicine, Tokyo Medical University, Tokyo, Japan; 3 Diamantina Institute for Cancer, Immunology and Metabolic Medicine, University of Queensland, Woolloongabba 4102 Australia; 4 Department of Hematology, Japanese Red Cross; 5 Department of Cellular Immunology, Medinet Medical Institute, Setagaya Ku, Tokyo, 158 0095 Japan

**Keywords:** gamma delta T cells, V*γ*9V*δ*2 T cells, immunotherapy, clinical trial

## Abstract

**Background:**

Adoptive transfer of *ex vivo* expanded autologous V*γ*9V*δ*2 T cells may be of therapeutic benefit for cancer because of their potent direct cytotoxicity towards tumour cells, synergistic cytotoxicity when combined with aminobisphosphonates and enhancement of antibody-dependent cell-mediated cytotoxicity.

**Methods:**

To determine the feasibility and clinical safety of therapy with *ex vivo* expanded, activated V*γ*9V*δ*2 T cells in combination with zoledronate, we enrolled 18 subjects with advanced solid tumours into a phase I clinical study. Administered indium^111^-oxine-labelled V*γ*9V*δ*2 T cells were tracked in a cohort of patients.

**Results:**

Administered V*γ*9V*δ*2 T cells had an activated effector memory phenotype, expressed chemokine receptors predictive of homing to peripheral tissues and were cytotoxic *in vitro* against tumour targets. Adoptively transferred V*γ*9V*δ*2 T cells trafficked predominantly to the lungs, liver and spleen and, in some patients, to metastatic tumour sites outside these organs. No dose-limiting toxicity was observed, but most patients progressed on study therapy. However, three patients administered V*γ*9V*δ*2 T cells while continuing previously ineffective therapy had disease responses, suggesting an additive effect.

**Conclusion:**

Therapy with aminobisphosphonate-activated V*γ*9V*δ*2 T cells is feasible and well tolerated, but therapeutic benefits appear only likely when used in combination with other therapies.

The majority of gamma/delta (*γδ*) T cells in human peripheral blood are of the V*γ*9V*δ*2 phenotype and constitute 1–5% of circulating lymphocytes (Carding and Egan, 2002; Chen and Letvin, 2003). Many *in vitro* and *in vivo* studies have demonstrated anti-tumour activity of V*γ*9V*δ*2 T cells, including against renal, prostate, colon and pancreatic cancers and melanoma, myeloma and lymphoma (Kabelitz *et al*, 2004, 2007; Bonneville and Scotet, 2006; Dieli *et al*, 2007; Bennouna *et al*, 2008; Abe *et al*, 2009). Mechanisms of anti-tumour activity include direct MHC non-restricted killing of tumour cells, antibody-dependent cell-mediated cytotoxicity (ADCC) (Lanier *et al*, 1985; Braakman *et al*, 1992; Tokuyama *et al*, 2008) and indirectly through activation of other immune effectors. There is evidence that suggests that *γδ* T cell cytotoxicity against a range of tumour cell lines is greater than that achievable with alpha/beta (*αβ*) T cells, which have been the focus of most trials of cancer vaccines and adoptive immune therapy (Ensslin and Formby, 1991; Lopez *et al*, 2000; Guo *et al*, 2005; Liu *et al*, 2005).

V*γ*9V*δ*2 T cells have a unique capacity to recognise and be activated and expanded by non-peptide phosphoantigens, including aminobisphosphonate drugs, such as zoledronate and pamidronate. Zoledronate has potent activity in activating and expanding V*γ*9V*δ*2 T cells, especially in combination with IL-2 (Sato *et al*, 2005; Kondo *et al*, 2008), and also sensitises tumour cells to V*γ*9V*δ*2 T cell cytotoxicity *in vitro* (Gober *et al*, 2003; Sato *et al*, 2005; Marten *et al*, 2007; Mattarollo *et al*, 2007; Bouet-Toussaint *et al*, 2008; Todaro *et al*, 2009) and *in vivo* in macaque (Gertner-Dardenne *et al*, 2009), in addition to having potential direct and indirect anti-tumour effects, including apoptotic and anti-proliferative functions (Senaratne *et al*, 2000; Tassone *et al*, 2000; Marten *et al*, 2007). Zoledronate indirectly inhibits MMP-9 and VEGF that are associated with tumour progression and invasion (Santini *et al*, 2003; Giraudo *et al*, 2004). This occurs through inhibition of the enzyme farnesyl pyrophosphate (FPP) synthase of the cellular mevalonate pathway, causing accumulation of isoprenoids, such as isopentenyl pyrophosphate (IPP), which stimulates and activates *γδ* T cells (Gober *et al*, 2003; Green *et al*, 2004). Of the bisphosphonates tested *in vitro*, zoledronate is the most potent inhibitor of FPP synthase (Clezardin, 2005; Knight *et al*, 2005). These properties make zoledronate a particularly interesting candidate for use in therapy aiming to harness the anti-tumour activities of *γδ* T cells (Das *et al*, 2001; Kato *et al*, 2001; Gober *et al*, 2003; Sato *et al*, 2005; Kondo *et al*, 2008).

Adoptive transfer of *ex vivo* expanded *γδ* T cells (Kobayashi *et al*, 2007; Bennouna *et al*, 2008; Abe *et al*, 2009; Noguchi *et al*, 2011) and *in vivo* therapeutic manipulation of *γδ* T cells by phosphoantigens and aminobisphosphonates with low-dose IL-2, have been reported and demonstrate potential anti-tumour activities of *γδ* T cells in patients with lymphoid malignancies (Wilhelm *et al*, 2003) and prostate cancer (Dieli *et al*, 2007). The combination of intravenous pamidronate or zoledronate and IL-2 for lymphoma, myeloma and prostate cancer was generally well tolerated but with side effects including fever, chills and transient flu-like symptoms (Wilhelm *et al*, 2003; Dieli *et al*, 2007) Adoptively transferred *ex vivo* expanded autologous V*γ*9V*δ*2 T cells with IL-2 also have limited toxicity, the most frequent adverse event being fever and general fatigue (Kobayashi *et al*, 2007; Bennouna *et al*, 2008; Abe *et al*, 2009). The toxicity of V*γ*9V*δ*2 T cells expanded with zoledronate and adoptively transferred in combination with a zoledronate infusion is yet to be reported in solid tumour patients.

Although healthy donor V*γ*9V*δ*2 T cells expand massively when stimulated *in vitro* by IL-2 in combination with phosphoantigens or bisphosphonates (Wilhelm *et al*, 2003), the *in vitro* proliferative capacity of V*γ*9V*δ*2 T cells from patients with malignancy seems less reproducible. For example, *in vitro* expansion of V*γ*9V*δ*2 T cells was poor in 50% of lymphoma patients (Wilhelm *et al*, 2003) and in 25% of renal carcinoma patients (Viey *et al*, 2005). It is unknown whether this defect in V*γ*9V*δ*2 T cell proliferation is tumour specific or broadly associated with malignancy, and it is also unknown whether the differences are directly related to differences in V*γ*9V*δ*2 T cells or due to other cellular constituents in the expansion cultures evaluated.

Adoptive transfer of V*γ*9V*δ*2 T cells as a therapeutic modality has a number of distinct advantages over active immune therapy with vaccines and direct stimulation of V*γ*9V*δ*2 T cells *in vivo* with either pharmaceutical agents or vaccines, but can also be seen as an additional mode of therapy with its own unique set of roles, rather than simply as an alternative to active immune therapy. For example, V*γ*9V*δ*2 T cells can be adoptively transferred directly after chemotherapy or tumour-targeting monoclonal antibodies, allowing infused cells to provide potential additive or synergistic cytotoxicity timed to coincide with the cytotoxic effects of the pharmaceutical and, in the case of monoclonal antibodies, to provide the potential for enhanced ADCC through CD16/FcR expression of infused cytotoxic cells (Mattarollo *et al*, 2007; Tokuyama *et al*, 2008). In contrast, it is difficult to time the maximal activity of cells expanded with vaccines, and chemotherapy administered after vaccination may inhibit proliferation of the desired cell population. The use of IL-2 to expand V*γ*9V*δ*2 T cells *in vivo* has the disadvantage of substantial IL-2 toxicity and the potential for expansion of regulatory T cells (Tregs), which may inhibit anti-tumour immunity (although both of these latter advantages are negated if IL-2 is used after adoptive therapy of V*γ*9V*δ*2 T cells).

In order for adoptively transferred V*γ*9V*δ*2 T cells to have a therapeutic role, they must traffic to tumour sites. Tumour-infiltrating lymphocytes include *γδ* T cells in many types of cancer, including colorectal, breast, prostate, ovarian and renal cell carcinomas, suggesting that these cells do have the capacity to infiltrate the tumour environment (Kabelitz *et al*, 2007). No clinical study has yet addressed the migration pattern of adoptively transferred V*γ*9V*δ*2 T cells in humans.

In the clinical study described here, the safety and feasibility of adoptive transfer of large numbers of *ex vivo* expanded autologous V*γ*9V*δ*2 T cells in combination with zoledronate infusion was investigated in patients with solid tumours. Evaluation of the destination of adoptively transferred V*γ*9V*δ*2 T cells is also reported. The *in vivo* effects of zoledronate on V*γ*9V*δ*2 T cells in peripheral blood were evaluated and localisation of adoptively transferred V*γ*9V*δ*2 T cells was assessed.

## Materials and methods

### Patients and healthy donors

Patients (*n*=18) and healthy donors (for *in vitro* studies only, *n*=10) were enrolled after providing informed consent. Patients with malignancy were enrolled for *in vitro* studies alone (*n*=27) or for *in vitro* studies plus therapy with V*γ*9V*δ*2 T cells and zoledronate (*n*=18). Patients enrolled had a range of metastatic solid tumours unresponsive to other therapies. Patient characteristics are summarised in Table 1. The study was approved by the Human Research Ethics Committee of the Greenslopes Private Hospital (Queensland, Australia).

### Treatment protocols

Initially, six patients were treated with a dose-escalation protocol (group A). The planned dose-escalation range of V*γ*9V*δ*2 T cells was 0.5 × 10^7^ to 500 × 10^7^; however, the maximum dose achieved in this initial group was 280 × 10^7^. Subsequently, nine patients received V*γ*9V*δ*2 T cells at an approximately fixed dose dependent on the proliferative capacity of their cells (group B). Each dose was generated from 1/8 of a single leukapheresis. Three additional patients received V*γ*9V*δ*2 T cells while continuing previous therapies that had not induced disease responses, but which were well tolerated (chemotherapy in two cases and hormonal therapy in one case) (group C).

Initial findings from the first three patients in the dose-escalation phase of the study indicated that zoledronate pretreatment substantially reduced V*γ*9V*δ*2 T cell number and expansion capacity. Therefore, the following 15 subjects underwent leukapheresis procedures before zoledronate administration and this initial dose was omitted. The zoledronate dose was split to administer a first dose (1 mg per treatment) 24 h before cell administration and a second 1 mg dose immediately before cell administration. This was based on our *in vitro* time course studies showing that zoledronate gives rapid but transient tumour sensitisation to V*γ*9V*δ*2 T cell killing in some cases, but that 24 h was required before maximal tumour sensitisation develops in other cases (Mattarollo *et al*, 2007).

### Clinical responses

Computed tomography (CT) scanning was used to evaluate treatment response. Complete remission (CR), partial remission (PR), stable disease (SD) or progressive disease (PD) were determined based on the RECIST criteria (Response Evaluation Criteria In Solid Tumors) (Therasse *et al*, 2000). During treatment, symptoms, clinical evaluation and haematological and biochemical evaluation of blood were used to monitor adverse events.

### Immunological monitoring

The following monoclonal antibodies for evaluating cell phenotypes using flow cytometry were obtained from Beckman Coulter (Fullerton, CA, USA): CD3 (UCHT1), CD4 (13B8.2), CD8 (SFCI21Thy2D3), CD69 (TP1.55.3), CD56 (N901), CD27 (1A4CD27), CD45RA (2H4LDH11LDB9), CD45 (J.33), TCR-Vγ9 (IMMU360), TCR-V*δ*2 (IMMU389) and TCR-pan *γ*/*δ* (IMMU510). Monoclonal chemokine receptor antibodies CCR5 (CTC5), CCR7 (150 503), CXCR3 (49 801) and CXCR5 (51 505.111) were obtained from R&D Systems Inc. (Minneapolis, MN, USA). Cell number was assessed by addition of flow-count beads (Beckman Coulter), and cell viability was determined by exclusion with 7-AAD (BD Biosciences, San Jose, CA, USA). Cells were stained according to the manufacturers' recommendations. All flow-cytometric analyses were performed using the Coulter Cytomics FC500 five-colour flow cytometer (Beckman Coulter).

### Proliferation and preparation of V*γ*9V*δ*2 T cells

Cells for adoptive transfer were generated under good manufacturing practice (GMP) conditions in purpose-built GMP laboratories within the University of Queensland laboratories at the Greenslopes Private Hospital. Peripheral blood mononuclear cells (PBMCs) were isolated by density gradient centrifugation using Ficoll-Paque (GE Healthcare, Buckinghamshire, UK) and V*γ*9V*δ*2 T cells selectively proliferated by culture of PBMCs in RPMI 1640 media (Lonza, Walkersville, MD, USA) supplemented with 10% human AB plasma (Lonza), L-glutamine (2 mM; Lonza) and gentamycin (40 *μ*g; Pfizer, Bentley, WA, Australia). Recombinant human IL-2 (700 IU ml^−1^; Novartis, Basel, Switzerland) and zoledronate (1 *μ*M; Novartis) were added on day 0 and additional IL-2 (350 IU ml^−1^) was added every 2–3 days during the culture period. After 7–14 days culture, purified effector cell populations containing 70–95% V*γ*9V*δ*2 T cells were obtained for *in vitro* functional assessment by depletion of CD4^+^, CD8^+^ and CD56^+^ cells using miniMACS (Miltenyi Biotec, Bergisch Gladbach, Germany). Cell populations for adoptive transfer were not purified, but were enriched by the culture procedure. The percentage of adoptively transferred cells that were V*γ*9V*δ*2 T cells is shown in Table 1.

### *In vitro* cytotoxicity assessment of V*γ*9V*δ*2 T cells

Cancer cell lines HT-29 and DLD-1 (colorectal), NCI-H358 (lung), TSU-Pr1 (bladder), DU-145 (prostate) and MDA-MB231 (breast) were used as representative solid tumour targets for functional assessment of V*γ*9V*δ*2 T cells. These cells were obtained from the American Type Culture Collection (ATCC; Manassas, VA, USA). All cell lines were cultured in RPMI 1640 media supplemented with 10% FCS, L-glutamine and gentamycin and maintained at 37 °C in 5% CO_2_. Adherent cells were detached using 0.05 M EDTA.

The cytotoxicity of V*γ*9V*δ*2 T cells against tumour targets was determined using the tetrazolium-based (MTS) assay referred to as CellTiter 96 (Promega, Madison, WI, USA) as described previously (Mattarollo *et al*, 2007). Purified V*γ*9V*δ*2 T cells were co-cultured with tumour targets at an effector/target ratio of 5 : 1 for a 4 h period at 37 °C. After co-culture, non-adherent effector cells and non-viable targets were removed and replaced with the MTS tetrazolium salt. After 4 h incubation, optical density values were read directly at 492 nm using the Multiskan Ascent microplate reader (Thermo, Vantaa, Finland). The viability of target cells was calculated as a percentage of the non-treated target control.

### Radiolabelling and clinical gamma (*γ)* camera imaging of V*γ*9V*δ*2 T cell distribution

Using radioactive indium^111^ oxine (In^111^)-labelled autologous V*γ*9V*δ*2 T cells, the migration of infused *ex vivo* expanded V*γ*9V*δ*2 T cells was evaluated in three patients (A2, B1 and B7). V*γ*9V*δ*2 T cells (1 × 10^8^) harvested from *in vitro* culture were labelled by incubation with 20 MBq of commercially prepared In^111^ (GE Healthcare) for 15 min, followed by two washes to remove any residual In^111^. Labelled cells were resuspended in saline and the radioactivity of the patient dose recorded (Atomlab 300 dose calibrator, Biodex, Shirley, NY, USA) and was in the range of 12–17 MBq. Patients received an infusion of 5 × 10^7^-labelled V*γ*9V*δ*2 T cells through a peripheral intravenous (IV) line. Full-body *γ*-imaging was performed using a dual-headed *γ*-camera (20% energy windows ∼173 and 247 keV) to monitor the location of labelled V*γ*9V*δ*2 T cells serially for 48 h starting within 30 min of injection (*t*=0 h) and then at 1, 4, 8, 24 and 48 h after injection. Cell accumulation in different organs was scored on a scale from 0 to 4: 0=no accumulation, 1=minimal accumulation, 2=low accumulation, 3=medium accumulation and 4=large accumulation.

### Statistical analysis

All statistical analyses were performed using Student's *t*-test and results were considered significant if *P*<0.05.

## Results

### Phenotype and cytotoxic activity of *in vitro* expanded V*γ*9V*δ*2 T cells

**Figure 1:**
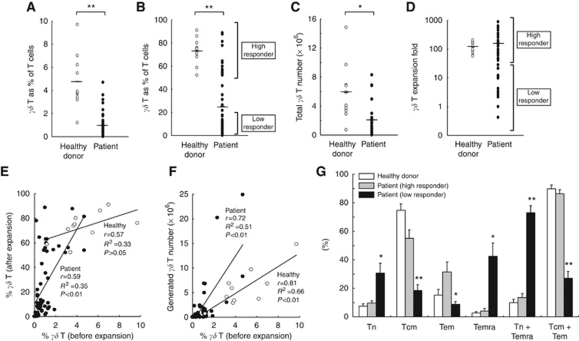
Proliferation and memory profiles of V*γ*9V*δ*2 T cells cultured *in vitro* with zoledronate and IL-2 for 14 days (*n*=10 healthy donors and *n*=45 patients with solid tumours). (**A** and **B**) The percentage of V*γ*9V*δ*2 T cells from peripheral blood of healthy donors and patients before and after *in vitro* culture. (**C** and **D**) V*γ*9V*δ*2 T cell numbers generated from 1 × 10^6^ healthy donor and patient PBMCs cultured *in vitro* and the expansion capacity. (**E** and **F**) Correlation between pre-culture V*γ*9V*δ*2 T cell percentages and post-culture V*γ*9V*δ*2 T cell percentages, expansion fold and absolute cell numbers. Hollow dots=healthy donors; solid dots=patients. (**G**) Naive (Tn), central memory (Tcm), effector memory (Tem) and CD45RA^+^ effector memory (Temra) V*γ*9V*δ*2 T cell memory subset profiles of pre-cultured healthy donor PBMCs (*n*=5) compared with low (*n*=10) and high (*n*=10) responding patient PBMCs (mean+s.e.m., ^**^P<0.01, ^*^P<0.05) (*γδ* T=V*γ*9V*δ*2 T cells).

The characteristics of V*γ*9V*δ*2 T cells in peripheral blood and after *in vitro* expansion for healthy donors (*n*=10) and patients with active, metastatic cancer (*n*=45) are summarised in Figure 1. Additional details for the subset of cancer patients (*n*=18) involved in the treatment phase of this study are summarised in Table 1.

In comparison with healthy donors, patients with active cancer, as a group, had significantly lower V*γ*9V*δ*2 T cells as a percentage of T cells (*P*<0.01; Figure 1A), lower percentages of V*γ*9V*δ*2 T cells in a potential therapeutic product after culture (*P*<0.01; Figure 1B) and lower numbers of V*γ*9V*δ*2 T cells that could be generated from a starting point of 1 × 10^6^ PBMCs (*P*<0.05; Figure 1C). Although the expansion capacity of V*γ*9V*δ*2 T cells was similar for cancer patients as a group compared with healthy individuals (Figure 1D), the cancer patient population was highly variable with respect to the expansion potential of V*γ*9V*δ*2 T cell numbers, and this is reflected in substantial variation in the purity of the potential V*γ*9V*δ*2 T cell therapeutic product (Figure 1B) and the total number of V*γ*9V*δ*2 T cells that could be generated (Figure 1C). The percentage of T cells that were V*γ*9V*δ*2 T cells before expansion culture predicted for both the purity of the final product (Figure 1E) and the total number of V*γ*9V*δ*2 T cells that could be generated (Figure 1F). The relative number of V*γ*9V*δ*2 T cells from different subsets before culture was predictive for V*γ*9V*δ*2 T cell expansion capacity, with high numbers of Tn+Temra predicting for poor expansion (Figure 1G) and the patient subgroups, defined as high and low responders, as shown in Figure 1B and D, differing with respect to their V*γ*9V*δ*2 T cell subset profile.

**Figure 2:**
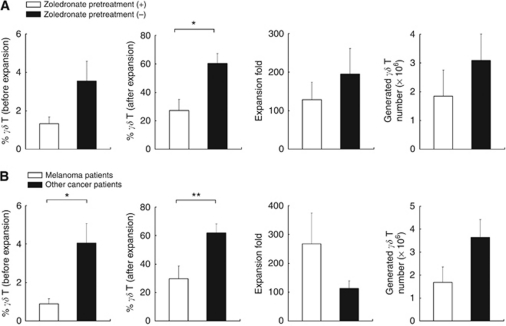
Proliferation of V*γ*9V*δ*2 T cells cultured *in vitro* with zoledronate and IL-2 for 14 days. Results of total cell numbers obtained are derived from a fixed starting number of PBMCs. (**A**) Previous treatment with zoledronate (*n*=6) *vs* no previous treatment with zoledronate (*n*=12). (**B**) Melanoma patients (*n*=7) *vs* other cancer patients (*n*=11) (mean+s.e.m., ^**^P<0.01, ^*^P<0.05) (*γδ* T=V*γ*9V*δ*2 T cells).

Patient clinical factors found to predict for the capacity to generate large numbers of V*γ*9V*δ*2 T cells included whether there had been previous treatment with zoledronate (Figure 2A) and tumour type (melanoma *vs* non-melanoma, Figure 2B). Patients who had received zoledronate at any time before collection of blood samples had lower initial V*γ*9V*δ*2 T cell numbers, less V*γ*9V*δ*2 T cells as a fraction of all lymphocytes, lower proliferative potential and a lower final number of V*γ*9V*δ*2 T cells achievable (Figure 2A). As a group, patients with melanoma had lower initial V*γ*9V*δ*2 T cells and lower final numbers of V*γ*9V*δ*2 T cells generated, despite no patients having been exposed to zoledronate (Figure 2B). The lack of exposure to zoledronate may explain the greater proliferative capacity of melanoma patients, as a group, than patients with other malignancies of whom many had received zoledronate.

**Figure 3:**
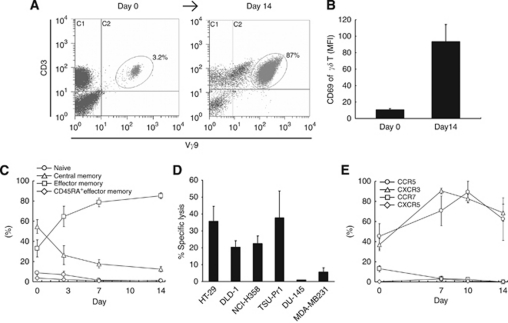
Phenotype and functional activity of *ex vivo* expanded patient V*γ*9V*δ*2 T cells. (**A**) Representative flow cytometry dot plot showing selective expansion of V*γ*9V*δ*2 T cells after 14 days culture in zoledronate and IL-2. (**B**) CD69 expression on V*γ*9V*δ*2 T cells (MFI value) after *in vitro* culture (mean±s.e.m.; *n*=10). (**C**) Relative proportions of V*γ*9V*δ*2 T cell subsets during the *in vitro* culture period (mean±s.e.m.; *n*=7). (**D**) Cytotoxicity of expanded V*γ*9V*δ*2 T cells against various solid tumour cell lines (mean±s.e.m.). Means are derived from separate killing assays using V*γ*9V*δ*2 T cells from 3 to 5 subjects. (**E**) Chemokine receptor profiles (peripheral-homing CCR5 and CXCR3; lymph node-homing CCR7 and CXCR5) of V*γ*9V*δ*2 T cells during *in vitro* culture (mean±s.e.m.; *n*=4) (*γδ* T=V*γ*9V*δ*2 T cells).

Our method of *in vitro* culture generated V*γ*9V*δ*2 T cells with purity of up to 90% after 14 days (without an additional purification step, Figure 1B and 3A). CD69 expression on V*γ*9V*δ*2 T cells increased significantly after *in vitro* expansion, indicating an activated phenotype (Figure 3B). The majority of the expanded V*γ*9V*δ*2 T cell population from normal donors and patients in the high-responder group were of the effector memory (Tem) and of the central memory (Tcm) phenotype (Figure 3C). Correlating with the effector phenotype observed using surface marker analysis, cultured V*γ*9V*δ*2 T cells were cytotoxic against a range of solid tumour cell lines *in vitro*, including HT29, DLD-1, NCI-H358 and TSU-Pr1 (Figure 3D). *In vitro* expanded V*γ*9V*δ*2 T cells had upregulated expression of peripheral tissue-homing chemokine receptors, CCR5 and CXCR3. In contrast, expression of lymphoid-homing receptors, CCR7 and CXCR5, decreased to undetectable levels (Figure 3E). These results show that V*γ*9V*δ*2 T cells expanded *in vitro* from cancer patients have effector cell characteristics including the capacity to effectively kill tumour targets and chemokine receptor expression profiles, suggesting the potential to migrate to peripheral tumour sites, although potentially not to disease-involved lymph nodes.

The population of cells infused to patients was predominantly V*γ*9V*δ*2 T cells as shown in Table 1. The other cells infused (expressed as a mean, s.d. and range) included NK cells (18±22%, range 0.5–75%) CD4^+^ T cells (6±6%, range 0.5–24%) and CD8^+^ T cells (17±16%, range 2–64%). We did not directly evaluate for co-expression of the V*γ*9V*δ*2 T cell receptor and CD56 (as separate analysis tubes were used), but V*γ*9V*δ*2 T cells are distinguishable in the context of these cultured cells from most *αβ* T cells (either CD4^+^ or CD8^+^) as they are CD3 positive but double negative for CD4 and CD8. The majority of cells administered were both V*γ*9V*δ*2 TCR positive and CD4CD8 double negative. With these caveats, our results indicate that a substantial proportion (about half) of the administered V*γ*9V*δ*2 T cells are CD56^+^ (49±23%, mean±s.d., range 27–73%).

### Clinical outcomes of V*γ*9V*δ*2 T cell administration

Details of administered V*γ*9V*δ*2 T cells, including cell numbers, purity, possible treatment-related adverse effects and clinical outcomes are summarised in Table 1. For preliminary assessment of safety and maximal tolerated doses of intravenous infusion of *in vitro* expanded V*γ*9V*δ*2 T cells, six patients (four melanoma, one ovarian cancer, one colon cancer) were treated with escalating doses of V*γ*9V*δ*2 T cells. In this group, the maximum V*γ*9V*δ*2 T cell dose per injection ranged from 0.04 × 10^9^ to 2.8 × 10^9^. The total accumulated dose of V*γ*9V*δ*2 T cells over the treatment period ranged from 0.1 × 10^9^ to 5.5 × 10^9^ with a mean of 2.8 × 10^9^. Maximal single dose and total dose were much lower than those predicted from the preclinical evaluation (before zoledronate administration) in the first three subjects because of markedly reduced expansion of V*γ*9V*δ*2 T cells from harvests undertaken after zoledronate administration.

The maximum dose of V*γ*9V*δ*2 T cells per treatment in the subsequent non-dose-escalation protocol (*n*=12) ranged from 0.3 × 10^9^ to 2.2 × 10^9^. The total doses of V*γ*9V*δ*2 T cells administered ranged from 1.0 × 10^9^ to 7.2 × 10^9^ with a mean of 2.8 × 10^9^, from a total of 6–8 treatments. Three patients (two breast cancer and one cervical) were treated with concurrent use of other therapy (two with chemotherapy and one with hormone therapy).

Of the 18 patients treated, 7 had fevers above 38 °C believed to be related to study therapy. Overall, side effects were manageable, tolerated by patients, did not interfere with their treatment and resolved within 24 h.

### Treatment outcome

Clinical response to treatment is summarised in Table 1. In the group of 15 patients with advanced cancer treated only with V*γ*9V*δ*2 T cells plus zoledronate, 3 had SD while 12 patients had PD during the study period. Although difficult to assess with their generally poor outcomes, there seemed no correlation between maximum or total dose of V*γ*9V*δ*2 T cells and clinical outcome. For example, patient A2 had large bulky disease, progressing before enrolment, but was stable during treatment and for a prolonged period after therapy was completed.

Three additional patients were assessed for treatment outcomes although their V*γ*9V*δ*2 T cells were administered in parallel with other therapies. All three of these patients experienced at least a partial response and one (breast cancer) had a complete response. Although it is not possible to know whether V*γ*9V*δ*2 T cells contributed to these responses, inclusion criteria dictated that subjects enrolled in our study were considered unlikely to respond to standard therapy. For example, patient C2 with breast cancer was non-responsive to hormonal therapy alone but had CR when hormonal therapy was combined with V*γ*9V*δ*2 T cell/zoledronate infusions. Patient C1 had PD when treated with chemotherapy alone before enrolment but had a PR and substantial symptomatic improvement when V*γ*9V*δ*2 T cell/zoledronate infusions were added to the same chemotherapy protocol. Patient C3, who presented with a rapid and florid relapse soon after cessation of previous chemotherapy, had a partial response to the combination of further chemotherapy and V*γ*9V*δ*2 T cell/zoledronate infusions.

### *In vivo* distribution and tumour localisation of adoptively transferred V*γ*9V*δ*2 T cells

To establish the migratory pattern of V*γ*9V*δ*2 T cells *in vivo*, trafficking of *in vitro* expanded V*γ*9V*δ*2 T cells was investigated in a cohort of patients (*n*=3).

In all patients administered In^111^-labelled V*γ*9V*δ*2 T cells (patients A2, B1, B7, 5 × 10^7^ V*γ*9V*δ*2 T cells per dose), rapid migration to the lungs occurred, where cells remained for 4–7 h. During this period, cell numbers (according to *γ*-activity) in the lungs then slowly decreased with gradual migration into the liver and spleen. Our previous studies have shown that this pattern does not relate to movement of free indium that might be released from cells after administration (Nieda *et al*, 2004). After 24 h, almost all cells were located in the liver and spleen and virtually no activity remained in the lungs. Although the timing of cell migration varied slightly, this pattern was consistent for all patients. Predominant accumulation of radioactivity in the lung, liver and spleen was consistent with a previous observation investigating initial localisation of tumour-specific *αβ* T cells (Meidenbauer *et al*, 2003) and there was no blood pooling (as indicated by the absence of significant radioactivity within the cardiac shadow), indicating egress of V*γ*9V*δ*2 T cells from the circulating blood pool. Repeat treatments with labelled V*γ*9V*δ*2 T cells gave the same pattern in all patients assessed more than once, including subjects B1 and A2 described below.

Assessment of the number of peripheral blood V*γ*9V*δ*2 T cells at multiple time points in the 48 h after V*γ*9V*δ*2 T cell infusion, mirroring the time points at which we evaluated V*γ*9V*δ*2 T cell by indium labelling, showed no substantial change in the number of peripheral blood V*γ*9V*δ*2 T cells compared with pre-infusion levels. These data are consistent with the nuclear medicine data indicating that few of the V*γ*9V*δ*2 T cells remain in the bloodstream.

**Figure 4:**
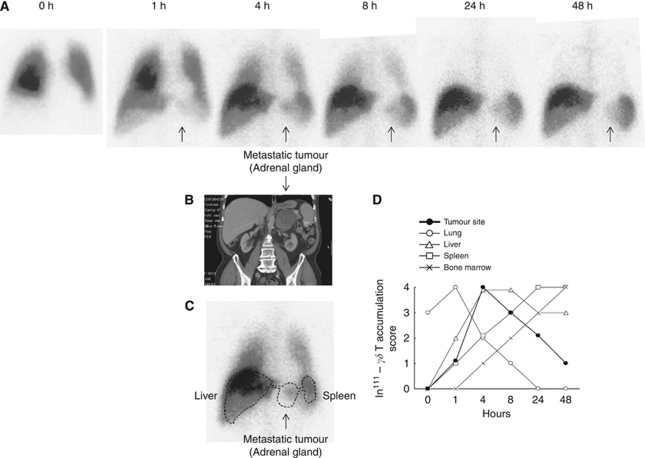
Localisation of adoptively transferred *in vitro* expanded V*γ*9V*δ*2 T cells. (**A**) Anterior *γ*-camera images of the chest and abdomen at various time points after In^111^-labelled V*γ*9V*δ*2 T cell transfer (5 × 10^7^ cells). Arrows point to location of tumour. (**B** and **C**) Abdominal CT scan (**B**) showing the tumour mass, the outline of which is superimposed onto the 4 h *γ*-image (**C**) to show V*γ*9V*δ*2 T cell activity at the tumour site. (**D**) Accumulation of V*γ*9V*δ*2 T cells in different organs over time, scored on a scale of 0 to 4, as described in the ‘Materials and Methods’ section (*γδ* T=V*γ*9V*δ*2 T cells).

In patient B1, who presented with an 84 × 57 × 75 mm^3^ metastatic mass on the left adrenal gland, a proportion of In^111^-labelled V*γ*9V*δ*2 T cells appear to have migrated to the tumour by 1 h after infusion. Maximal activity was seen in the tumour area at 4 h and tracer (and presumably intact cells) remained in the metastatic tumour site for the remainder of the 48 h observation period (Figure 4A–C). Accumulation of labelled cells in the tumour followed a different time course to that in other organs (Figure 4D). In patient A2, who presented with 89 mm and 26 mm tumours in the left lung, accumulation of labelled cells at the 89 mm tumour site was also observed at 4 h, but this activity was more subtle than for patient B1 (data not shown) suggesting lower numbers of cells within the tumour site.

## Discussion

The phase I trial described here indicates that combination therapy involving V*γ*9V*δ*2 T cells with zoledronate is feasible in patients with advanced solid tumours and is well tolerated. Our results highlight a number of practical issues that need addressing in future studies, including ways of increasing the proportion of patients who may benefit from this treatment, the need for more studies evaluating the destination of infused cells and the need to evaluate V*γ*9V*δ*2 T cells in combination with other therapies. Despite the technical demands of generating V*γ*9V*δ*2 T cells for adoptive therapy, adoptive therapy is ideal for evaluating combinations of V*γ*9V*δ*2 T cells with other therapeutic modalities as administration of V*γ*9V*δ*2 T cells can be appropriately timed to ensure maximal synergy and avoidance of chemotherapeutic damage to V*γ*9V*δ*2 T cells.

Our study confirms that *in vitro* generation of V*γ*9V*δ*2 T cells for adoptive therapy is achievable in many cancer patients, despite advanced disease or previous chemotherapy. However, to maximise the potential for a therapeutic benefit from V*γ*9V*δ*2 T cell adoptive immune therapy higher cell doses may be required. Minimal therapeutic benefits were observed at the doses used in our study. To evaluate potential benefits of higher cell doses, factors inhibiting V*γ*9V*δ*2 T cell number and *in vitro* expansion capacity in cancer patients need to be addressed. Furthermore, ways to ensure V*γ*9V*δ*2 T cell survival (and possibly additional *in vivo* expansion), trafficking to tumour sites and retention of cytotoxic activity after infusion need to be explored.

In the initial stages of our study, depletion of circulating V*γ*9V*δ*2 T cell numbers and *in vitro* expansion capacity were repeatedly observed after a single dose of zoledronate (data not shown). The changes were so marked that a protocol change was necessitated, avoiding zoledronate administration before cell harvesting. Similar aminobisphosphonate-induced decreases in V*γ*9V*δ*2 T cell numbers have been reported in patients with prostate cancer (Dieli *et al*, 2007) and in primates (Cendron *et al*, 2007). In our patient samples, low baseline percentages of V*γ*9V*δ*2 T cells and increased proportions of Tn+Temra subsets (as a fraction of total pre-culture T cells) predicted for low numbers of V*γ*9V*δ*2 T cells achieved after culture (data not shown).

Regulatory T cells may also negatively regulate V*γ*9V*δ*2 T cell proliferation. Regulatory T cells dampen T cell immunity (Zou, 2006) and are known to inhibit immune responsiveness in patients with advanced malignancy (Gallimore and Godkin, 2008). The percentage of Tregs in the blood of melanoma patients is significantly higher than that for healthy donors (Cesana *et al*, 2006), and we observed poor V*γ*9V*δ*2 T cell expansion in melanoma patients in our study. Our preliminary data (unpublished observations) and previous reports (Kabelitz *et al*, 2007; Kunzmann *et al*, 2009) indicate that depletion of Tregs from patient mononuclear cells leads to greater V*γ*9V*δ*2 T cell expansion *in vitro*.

Depletion of Tregs, either *in vivo* before harvest or *in vitro* and cryopreservation of large numbers of PBMCs before aminobisphosphonates, may be a pre-requisite for successful utilisation of V*γ*9V*δ*2 T cells for adoptive cell therapy in subjects who may receive aminobisphosphonates as part of their standard therapy for bone disease or as part of an immunotherapeutic strategy.

Efficient migration into tumour sites of actively cytotoxic subsets is a prerequisite for adoptively transferred V*γ*9V*δ*2 T cells to be therapeutically useful in cancer treatment. We demonstrated that V*γ*9V*δ*2 T cells we infused, which contained a range of subsets, had cytotoxic activity *in vitro* and included a substantial proportion of V*γ*9V*δ*2 T cells predicted to have cytotoxicity by virtue of CD56 expression (Thedrez *et al*, 2009; Urban *et al*, 2009). However, as we did not select subsets for our trafficking studies or our cytotoxicity assays, we do not know whether those cells eliciting *in vitro* cytotoxicity were those that migrated to the tumour sites.

Increased effector memory *γδ* T cells are reported to correlate with objective clinical outcomes in patients treated with zoledronate and IL-2 (Dieli *et al*, 2007). The culture conditions we used generated large numbers of effector memory V*γ*9V*δ*2 T cells from the blood of cancer patients. In addition, as a surrogate assessment to predict the capacity for *in vitro* activated V*γ*9V*δ*2 T cells to migrate to tumour tissue, chemokine receptor expression was investigated. Expression of CCR5 and CXCR3 by effector T cells is reported to be important for trafficking to tumour sites as these receptors respond to a range of chemokines such as I-TAC, MIG, IP-10 (ligands for CXCR3) and RANTES, MIP-1*α*, MIP-1*β*, MCP-2 (ligands for CCR5) released by tumours and inflammatory tissue (Moser and Loetscher, 2001). V*γ*9V*δ*2 T cells from healthy donors are reported to express high constitutive levels of CCR5, but this is downregulated within 24–48 h of IPP stimulation in parallel with upregulation of the lymphoid-homing chemokine receptor, CCR7 (Glatzel *et al*, 2002; Brandes *et al*, 2003). Although a similar pattern of initial CCR5 downregulation and CCR7 upregulation was observed with our zoledronate- and IL-2-containing cultures (data not shown), after further V*γ*9V*δ*2 T cell expansion, there were large increases in both CCR5 and CXCR3 expression, peaking at days 7–10. Minimal changes in CCR7 and CXCR5 expression were observed, with levels remaining low at the end of the culture period.

The results of the trafficking studies with activated V*γ*9V*δ*2 T cells reported here indicate localisation of V*γ*9V*δ*2 T cells at a large tumour site from as early as 1 h after infusion, with maximal activity at 4 h and persistence for at least 48 h. Although only a minority of the administered cells localised to the tumour, this is the first direct demonstration of adoptively transferred V*γ*9V*δ*2 T cell localisation at a tumour site with concurrent use of zoledronate. Although these results are encouraging, further work is required to characterise the properties of V*γ*9V*δ*2 T cells that migrate to the tumour and to increase the proportion of V*γ*9V*δ*2 T cells that migrate to tumour sites. The overall migration pattern we observed, in which initial, transient retention in the lungs is followed by movement predominantly to the liver and spleen, has been observed previously for other lymphocyte populations and is not specific to V*γ*9V*δ*2 T cells (Fisher *et al*, 1989; Nieda *et al*, 2004). It is unknown to what extent accumulation of activity in the lungs, liver and spleen indicates active homing of viable V*γ*9V*δ*2 T cells rather than passive ‘trapping’. It is also unclear whether V*γ*9V*δ*2 T cells infiltrate the parenchyma (providing an opportunity for therapeutic benefits for tumours in these organs) or remain within the lumen of blood vessels. More sensitive imaging technology is required to answer these questions.

Evaluation of clinical responses was not a major end point of the trial but clearly no disease responses (other than stabilisation) were observed in the 15 patients treated with only the V*γ*9V*δ*2 T cell/zoledronate combination. However, of potential interest, was the observation of clinical responses in three of three patients in whom conventional therapy was used in parallel with V*γ*9V*δ*2 T cell/zoledronate therapy (one CR in combination with ongoing hormone therapy after failure of hormone therapy alone and two PR in combination with chemotherapy, including one patient with chemorefractory disease and another patient with very early relapse after previous chemotherapy). These clinical observations are consistent with *in vitro* studies indicating that the combination of V*γ*9V*δ*2 T cells with chemotherapy produces significantly greater tumour cell death than when either modality of treatment is used alone (Mattarollo *et al*, 2007). Of added interest are the previous *in vitro* observations of synergistic cytotoxic effects of CD16 expressing V*γ*9V*δ*2 T cells with therapeutic monoclonal antibodies (Tokuyama *et al*, 2008).

Previously, successful studies of adoptive immune therapy have included the use of IL-2 after cell therapy administration. The withdrawal of exposure to high concentrations of IL-2 used in the laboratory may limit the survival and function of V*γ*9V*δ*2 T cells after their infusion. We did not administer IL-2 to patients in our study to evaluate the toxicity profile of V*γ*9V*δ*2 T cells but this may have limited the potential for therapeutic benefits.

In summary, the combination of zoledronate and IL-2 generates large numbers of V*γ*9V*δ*2 T cells *in vitro* with cytotoxic activities against a range of tumour types, even in heavily pretreated patients with advanced malignancy. Administration of these cells is safe. Administered cells have a phenotype suggesting the potential to migrate to tumour tissues and we provide preliminary clinical evidence for migration of V*γ*9V*δ*2 T cells to tumour sites. As cells similar to those administered in this study have previously been shown to enhance the cytotoxic effects of chemotherapeutic agents and monoclonal antibodies, we propose further studies of zoledronate-activated V*γ*9V*δ*2 T cells in combination with chemotherapy and monoclonal antibodies.

## Figures and Tables

**Table 1:** Characteristics of patients, *ex vivo* expansion of patients' V*γ*9V*δ*2 T cells and treatment and clinical outcomes

						**% *γδ* T in CD3+** ^*^			***Ex vivo* expanded *γδ* T**					
**Patient**	**Age (years)/ sex**	**Primary cancer**	**Metastasis**	**Previous therapy**	**Previous Zol. treatment**	**Before expansion**	**After *ex vivo* expansion**	**Expansion fold**	**Treatments**	**Max. dose/ treatment ( × 10^9^cells)**	**Total dose ( × 10^9^cells)**	**Toxicity** a	**Clinical response**	**Comment**
*Group A (GDT dose escalation/Zol. treatment)*														
A1	58/F	Melanoma	Lung	—	Yes	0.4 (2.0)	8.9 (2.8)	28 (13)	8	0.04	0.1	Yes	PD	
A2	59/M	Melanoma	Lung	—	Yes	2.4 (3.0)	23.5 (4.0)	8 (2)	8	0.2	0.5	No	SD	—b
A3	66/F	Melanoma	Lung, liver	I	Yes	0.5 (0.7)	20.3 (4.8)	95 (24)	8	0.6	2.0	No	PD	
A4	60/F	Ovarian cancer	Peritoneum	C	No	5.7 (0.3)	62.3 (5.0)	34 (7)	8	1.5	3.5	No	SD	
A5	67/F	Melanoma	Abdomen	—	No	1.3 (0.7)	55.7 (4.3)	262 (81)	8	2.3	5.0	No	PD	—c
A6	56/F	Colon cancer	Lung, liver	C	No	11.1 (2.8)	85.8 (4.5)	47 (11)	8	2.8	5.5	Yes	PD	
														
*Group B (GDT non-dose escalation/Zol. treatment)*														
B1	67/M	Melanoma	Adrenal grand, heart	I	No	0.3 (0.1)	15.3 (2.2)	728 (111)	6	0.3	1.0	No	SD	
B2	48/F	Adenocarcinoma	Bone	R	No	2.1 (0.5)	53.6 (9.9)	144 (72)	8	0.5	1.1	Yes	PD	
B3	47/M	Cholangiocarcinoma	Local advanced disease	C	No	1.8 (0.1)	59.5 (4.8)	17 (2)	8	0.4	1.4	No	PD	
B4	65/F	Melanoma	Lung, abdominal mass	I	No	0.5 (0.1)	12.3 (1.9)	159 (84)	8	0.5	1.4	No	NE	
B5	61/F	Melanoma	Lung	—	No	0.8 (0.0)	71.4 (6.6)	586 (273)	7	1.0	1.7	No	PD	
B6	61/F	Ovarian carcinoma	Peritoneum	C	No	5.1 (0.7)	86.6 (2.0)	43 (7)	8	1.0	3.0	No	PD	
B7	51/F	Colon cancer	Lung, liver	C, R, I	No	2.6 (0.3)	70.0 (3.8)	86 (14)	8	0.8	3.3	Yes	PD	
B8	57/F	Colon cancer	Lung	C, R	Yes	2.3 (0.1)	64.0 (3.1)	253 (25)	6	1.5	4.6	No	PD	
B9	68/M	Duodenal cancer	Lung, abdomen	C	No	9.1 (0.4)	71.7 (3.9)	78 (13)	8	2.2	7.2	Yes	PD	
														
*Group C (GDT/Zol. treatment with other therapy)*														
C1	58/F	Breast cancer	Brain, liver, lung	C	Yes	1.3 (0.1)	22.4 (4.5)	119 (34)	7	0.3	0.9	No	PR	—d
C2	44/F	Breast cancer	Bone, liver	C, R, H	Yes	1.1 (0.1)	24.3 (5.7)	269 (143)	7	1.5	3.6	Yes	CR	—e
C3	33/F	Cervical cancer	Lung, pelvis	C	No	2.3 (1.0)	78.9 (6.9)	160 (32)	8	1.9	4.0	Yes	PR	—d

Abbreviations: C=chemotherapy; CR=complete remission; γδ T=V*γ*9V*δ*2 T cell; H=hormonal therapy; I=immunotherapy; inj.=injection; NE=not evaluable; PD=progressive disease; PR=partial remission; R=radiotherapy; S=surgery; SD=stable disease; Zol=Zoledronate;

^*^Represents the mean (s.e.) from 6–8 vaccines.

a Fever after infusion, A1 also had vomiting.

b Large bulk of disease but stable.

c No new lesions.

d With chemotherapy.

e With hormonal therapy.
